# Low Testosterone Level and Mortality Risk in Patients With Prostate Cancer: A Post‐Randomization Analysis

**DOI:** 10.1002/cam4.71124

**Published:** 2025-08-01

**Authors:** Sayeh Fattahi, Ming‐Hui Chen, Jing Wu, Alicia C. Smart, Anthony V. D'Amico

**Affiliations:** ^1^ Department of Radiation Oncology Brigham and Women's Hospital and Dana Farber Cancer Institute Boston Massachusetts USA; ^2^ Department of Statistics University of Connecticut Storrs Connecticut USA; ^3^ Department of Computer Science and Statistics University of Rhode Island Kingston Rhode Island USA

**Keywords:** mortality, prostate cancer, prostate‐specific antigen, testosterone, transgender

## Abstract

**Background:**

A low serum testosterone can prolong the time needed for PSA to exceed normal and prompt a work‐up to rule out prostate cancer (PC), delaying diagnosis. We evaluated PC aggressiveness at diagnosis and PC‐specific and all‐cause mortality (PCSM, ACM)‐risk within comorbidity subgroups in patients with low versus normal testosterone.

**Methods:**

Between 2005 and 2015, 350 PSA‐screened patients with tumor (T) category1c‐4N0M0 unfavorable‐risk PC were enrolled in a randomized trial and comprised the study cohort. Fine and Gray and Cox multivariable regression analyses were used to evaluate PCSM and ACM risk, respectively, adjusting for age, known PC prognostic factors, randomized treatment arm, and the time‐dependent use of salvage androgen deprivation therapy. An interaction term between the Adult Comorbidity Evaluation‐27 defined comorbidity and low versus normal testosterone was included in the models to enable an assessment of PCSM and ACM risk within comorbidity subgroups in patients with low versus normal testosterone levels at randomization.

**Results:**

After a median follow up of 10.20 years, 89 of 350 patients died (25.43%) with 42 of 89 deaths (47.19%) from PC. In patients with no or minimal but not moderate to severe comorbidity, a significant association was observed between low compared to normal testosterone level at randomization and increased PCSM (AHR: 2.70 [95% CI: 1.27, 5.76], *p* = 0.01) and ACM risk (AHR: 1.90 [95% CI: 1.11, 3.26], *p* = 0.02).

**Conclusion:**

Unlike PSA, multiparametric MRI (mpMRI) images are not influenced by the serum testosterone level; therefore, evaluating whether PCSM can be reduced by incorporating mpMRI into PC‐screening in otherwise healthy men or transgender women with a low serum testosterone level should be considered.

**Trial Registration:**

The statisical code used to derive the results from the interaction model for this post randomization analysis can be found in the Supporting Information.

## Graphical abstract

In this post‐randomization analysis, patients in otherwise good health with low versus normal testosterone had a significantly higher prostate cancer‐specific and all‐cause mortality risk. Investigating whether death from prostate cancer can be reduced by incorporating multiparametric MRI into prostate cancer screening in men or transgender women with low serum testosterone should be considered.

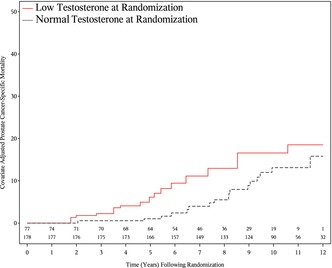

## 1 Introduction

Low serum testosterone is associated with more advanced prostate cancer (PC) at presentation and a higher risk of PC‐specific mortality (PCSM) [1, 2, 3, 4, 5, 6, 7, 8, 9]. This finding may be due to the inability to enable early detection when using a prostate specific antigen (PSA) cut‐point to recommend further work‐up to diagnose PC because patients with low serum testosterone do not mount the same PSA response as patients with normal serum testosterone, given that PSA expression is androgen dependent [10]. Low testosterone level is associated with shorter overall survival (OS) due to hormone‐related metabolic‐associated diseases such as metabolic syndrome, diabetes mellitus, and cardiovascular disease [11]. One study did not observe an association between patients with low serum testosterone and an increased PCSM and all‐cause mortality (ACM) risk [12]. However, in that study, clinical tumor (T)‐category and comorbidity at baseline, as well as use of salvage androgen deprivation therapy (ADT) at the time of PSA failure, were not adjusted for in the model. In addition, the testosterone level was subdivided into five categories as compared to two (low versus normal) which could have lowered the power of the study to observe the association.

Before 2020, to establish a diagnosis of PC, a standard 12‐core systematic transrectal ultrasound (TRUS)–guided biopsy of the prostate was used. Yet using this approach, clinically significant PC can be missed in 30.2% of cases due to biopsy sampling error [13]. With the contemporary use of multiparametric magnetic resonance imaging (mpMRI) to identify suspicious areas for targeted biopsy in people with an elevated PSA level, it has been shown that using a combined targeted and systematic 12‐core TRUS‐guided biopsy approach reduces the chance of missing clinically significant PC to 6.7% [13].

For people with a low serum testosterone level, the current PC screening guideline from the American Urologic Association recommends further workup in patients with mpMRI and biopsy only if the PSA is elevated [14]. Moreover, the digital rectal exam (DRE) which may reveal a prostate nodule in a person with low serum testosterone and a normal PSA level, is no longer recommended as part of routine screening in the primary care setting [15, 16, 17, 18, 19, 20, 21, 22]. However, waiting for men with a low serum testosterone or transgender women on gender affirming hormonal therapy (GAHT) who also have a low serum testosterone level to reach a PSA exceeding the upper limit of normal (ULN) to initiate a workup to rule out PC can lead to a more advanced stage at diagnosis and, as a result, a worse prognosis. Of note, in a recent study assessing the PSA level in transgender women on GAHT and not known to have PC, the median PSA (interquartile range [IQR]) was 0.02 ng/mL (< 0.01, 0.20), where 36% of the study cohort had an undetectable PSA and the maximum PSA was 2.21 ng/mL [23]. Therefore, the question of whether mpMRI should be performed as part of PC screening in people with a low serum testosterone to provide the possibility for earlier detection and potentially improve prognosis remains unknown and is clinically significant.

Therefore, to assess the need to prospectively investigate whether earlier detection of PC using mpMRI could reduce mortality‐based outcomes among patients with low testosterone, we used data from a prospective randomized trial where people were PSA‐screened to enumerate and compare clinical factors at presentation stratified by low versus normal testosterone. We further used prospectively ascertained follow‐up data to assess whether a low serum testosterone level at presentation was associated with an increased PCSM and ACM risk among patients with no or minimal versus moderate to severe comorbidity after adjusting for age and known PC prognostic factors including clinical T‐category at randomization and time‐dependent use of salvage ADT for PSA recurrence.

## 2 Methods

### 2.1 Patient Population, Staging, and Treatment

Between September 21, 2005, and January 13, 2015, 350 PSA‐screened patients from the United States, Australia, and New Zealand with PSA‐screen detected Tumor (T) category 1c‐4N0M0 unfavorable‐risk PC were enrolled in a randomized PC treatment trial (ClinicalTrials.gov Identifier: NCT00116142) where the two treatment arms were: (1) radiation therapy (RT) and ADT plus docetaxel, and (2) RT and ADT. Serum testosterone level was ascertained at enrollment for all but 19 patients. As a result, we categorized serum testosterone level into three groups: low, normal, and missing. We found that the risks of our endpoints (PCSM and ACM) were not significantly different between the normal and missing testosterone level groups and therefore combined these two groups. A low testosterone level was defined as a serum level < 264 ng/dL based on the Endocrine Society Clinical Practice Guideline [24] with a 20% margin to account for the daily and seasonal variations in serum testosterone levels [25, 26]. All patients underwent staging with bone scan and computerized tomography or MRI of the pelvis. Prostate biopsy specimens were reviewed by a pathologist with expertise in genitourinary pathology. Baseline patient data included demographics, comorbidities, prostate cancer prognostic factors, and clinical T‐category, and were collected before randomization. In accord with federal and institutional guidelines, patients signed a Dana Farber Harvard Cancer Center institutional review board (IRB)‐approved, protocol‐specific informed consent form permitting prospective collection of deidentified data at baseline and at follow‐up, which were entered into a secure, password‐protected database for outcome analysis at our institution. For the current study, the IRB deemed it to be exempt with respect to both obtaining consent and ethics approval.

### 2.2 Follow‐Up and Determination of Cause of Death

Following the end of RT, patients were seen for follow‐up every 6 months for 5 years and annually thereafter. At each follow‐up, serum PSA and testosterone levels were obtained. Follow‐up patient data also included overall survival status and date and cause of death if applicable. The cause of death was centrally reviewed by the principal investigator (AVD) who was blinded to the randomized treatment arm.

### 2.3 Statistical Methods

#### 2.3.1 Comparison of the Distribution of Clinical Factors Stratified by Low Versus Normal Testosterone

Descriptive statistics were used to characterize and compare the distribution of the clinical factors, including patient age and comorbidity at randomization, randomized treatment arm, and PC prognostic factors among patients with low versus a normal serum testosterone level. For the categorical covariates of T‐category (3b/4 vs. 2/3a vs. 1c), Gleason score (9–10 vs. 8 vs. 7 or less), Adult Comorbidity Evaluation (ACE)‐27 comorbidity metric (moderate to severe vs. no or minimal), PSA (< 4 vs. ≥ 4–10 vs. > 10–20 vs. > 20 ng/mL), and randomized treatment arm (RT + ADT + Docetaxel vs. RT + ADT), a Mantel–Haenszel Chi‐Square metric was used [27]. For the continuous covariate of age in years at randomization, a two‐sample Wilcoxon test was used [28].

#### 2.3.2 Prostate Cancer‐Specific and All‐Cause Mortality Adjusted Hazard Ratios

Fine and Grays [29] and Cox multivariable regression [30] analyses were used to evaluate the endpoints of PCSM and ACM, respectively. We used Fine and Grays competing risks regression [29] to evaluate the PCSM endpoint because 57.81% of all deaths were from non‐prostate cancer causes. Time zero was considered the date of randomization. We included an interaction term in our multivariable Cox [30] and Fine and Grays [29] regression models. This term is a product of two independent covariates being testosterone level (low versus normal) and ACE‐27 comorbidity level (moderate to severe versus no or minimal) at randomization. This interaction term allowed us to examine four effects which we detail further in a Supporting Information S1. The four effects included the impact on PCSM and ACM risk of a low versus normal testosterone level at randomization among patients with moderate to severe as well as no or minimal comorbidity and also allowed us to investigate the impact on PCSM and ACM risk of moderate to severe versus no or minimal comorbidity among patients with a normal or low testosterone level at randomization. An adjusted hazard ratio (AHR) with a 95% confidence interval (CI) was reported for each covariate assessed. Covariates included in the model were age at randomization (continuous), T‐category (3b/4 vs. 2/3a vs. 1c as baseline), Gleason score (9–10 vs. 8 vs. 7 or less as baseline), PSA (< 4 vs. ≥ 4–10 as baseline vs. > 10–20 vs. > 20 ng/mL), and randomized treatment arm (RT + ADT + docetaxel vs. RT + ADT as baseline). Salvage ADT at the time of PSA recurrence defined as nadir + 2 ng/mL was included in the models as a time‐dependent covariate.

#### 2.3.3 Adjusted Estimates of Prostate Cancer‐Specific and All‐Cause Mortality

**FIGURE 1:**
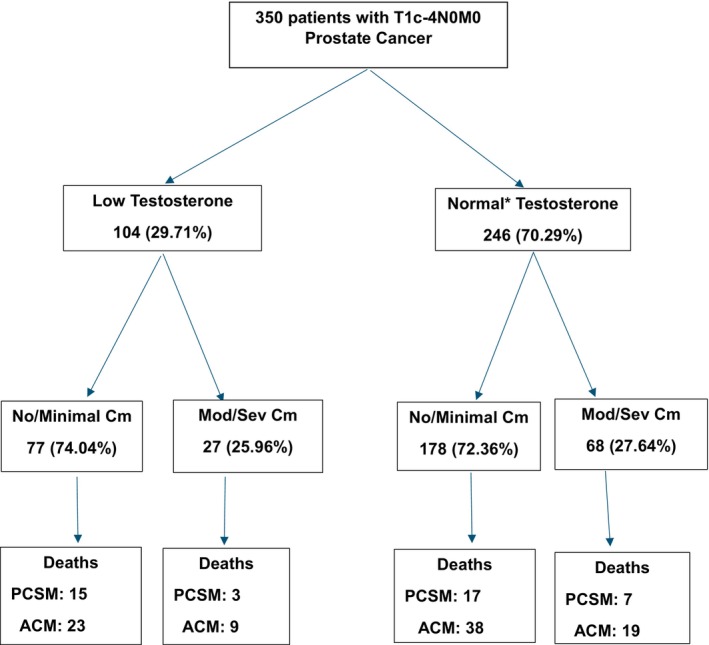
CONSORT diagram. ACM: all‐cause mortality; Cm: Adult Comorbidity Evaluation‐27; Mod/Sev: moderate/severe; PCSM: prostate cancer‐specific mortality; T: tumor category; Testosterone: serum testosterone. *Includes 19 patients with missing serum testosterone levels.

To illustrate the results of the multivariable models, adjusted cumulative incidence estimates [31] of PCSM and 1—Kaplan–Meier–adjusted estimates [32] of overall survival or ACM stratified by the ACE‐27 comorbidity level (moderate to severe, no or minimal) and serum testosterone level (low or normal at randomization) were calculated and compared, using the extended Kaplan–Meier methodology to adjust for the time‐dependent covariate of salvage ADT [33] and the weighted methodology to adjust for the other covariates in the multivariable models [34]. This resulted in four groups as shown in the CONSORT diagram in Figure 1. We applied a Bonferroni correction [35] to adjust for the four comparisons such that a two‐sided *p*‐value < 0.05/4 or < 0.0125 was considered statistically significant. We used Kaplan–Meier methodology [32] by reversing the censored indicator as the event indicator to calculate the median follow‐up. SAS version 9.4 (SAS Institute Inc., Cary, NC) was used for all calculations.

## 3 Results

### 3.1 Comparison of the Distribution of Clinical Factors Stratified by Low Versus Normal Testosterone

Table 1 shows a comparison of the distribution of clinical factors at randomization stratified by low versus normal testosterone. Patients with a low testosterone were significantly more likely (*p* = 0.04) to present with more advanced T‐category. There was no statistically significant difference in the distribution of Gleason score (9–10 vs. 8 vs. 7 or less) when stratified by low versus normal testosterone (*p* = 0.12). However, a near significant difference was observed when the Gleason score distribution was changed to 9–10 vs. 8 or less (*p* = 0.06) with Gleason score 9–10 being more likely in patients with a low serum testosterone.

**TABLE 1:** Comparison of the distribution of clinical factors at randomization stratified by low versus normal testosterone.

Clinical factor	Low testosterone median [IQR]	Normal testosterone median [IQR]	*p*
	*N* = 104	*N* = 246	
	243 ng/dL (182, 291)	484 ng/dL (375, 677)	
Tumor category			
3b/4	11 (10.58%)	22 (8.94%)	0.04
2/3a	75 (72.12%)	149 (60.57%)	
1c	18 (17.31%)	75 (30.49%)	
Gleason score			
9–10*	45 (43.27%)	80 (32.52%)	0.12
8	13 (12.50%)	29 (11.79%)	
7 or less	46 (44.23%)	137 (55.69%)	
PSA in ng/mL			
< 4	10 (9.62%)	17 (6.91%)	0.86
≥ 4–10	39 (37.50%)	95 (38.62%)	
> 10–20	28 (26.92%)	67 (27.24%)	
> 20	27 (25.96%)	67 (27.24%)	
Age (years) median (IQR)	67 (62, 72)	66 (61, 70)	0.18
Randomized treatment arm			
RT + ADT + docetaxel	59 (56.73%)	116 (47.15%)	0.10
RT + ADT	45 (43.27%)	130 (52.85%)	
ACE‐27 defined			
Moderate to severe comorbidity	27 (25.96%)	68 (27.64%)	0.75
No or minimal comorbidity	77 (74.04%)	178 (72.36%)	

Abbreviations: ACE: adult comorbidity evaluation; ADT: androgen deprivation therapy; IQR: interquartile range; ng/mL: nanograms/mL; PSA: prostate‐specific antigen; RT: radiation therapy.

* *p*‐value = 0.06 comparing Gleason score 9–10 versus Gleason score 8 or less.

### 3.2 Prostate Cancer‐Specific and All‐Cause Mortality Adjusted Hazard Ratios

After a median follow up of 10.20 years (IQR: 7.96, 11.41), 89 of 350 people died (25.43%), and of these deaths, 42 (47.19%) were from PC. In patients with no or minimal but not moderate to severe comorbidity, a significant association was observed between a low testosterone level and increased PCSM‐ (AHR: 2.70 [95% CI: 1.27, 5.76], *p* = 0.01) and ACM risk (AHR: 1.90 [95% CI: 1.11, 3.26], *p* = 0.02) when patients with a normal testosterone level at randomization served as the reference group, as shown in Table 2. Other factors associated with a significant increase in the risk of both PCSM and ACM included Gleason score 9–10 (PCSM AHR: 3.41 [95% CI: 1.73, 6.75], *p* < 0.001), ACM (AHR: 2.14 [95% CI: 1.34, 3.43], *p* = 0.002) and use of salvage ADT at the time of PSA failure (PCSM AHR: 19.41 [95% CI: 7.13, 52.84], *p* < 0.001), ACM (AHR: 2.83 [95% CI: 1.72, 4.63], *p* < 0.001).

**TABLE 2:** Multivariable interaction regression model adjusted hazard ratios for all‐cause and prostate cancer‐specific mortality for each clinical factor.

Clinical factor	No. of patients	ACM			PCSM		
		No. of deaths	AHR [95% CI]	*p*	No. of PC deaths	AHR [95% CI]	*p*
No or minimal ACE‐27 comorbidity testosterone in ng/dL							
Low	77	23	1.90 [1.11, 3.26]	0.02	15	2.70 [1.27, 5.76]	0.01
Normal	178	38	1.00 Ref	—	17	1.00 Ref	—
Moderate to severe ACE‐27 comorbidity testosterone in ng/dL							
Low	27	9	1.00 [0.43, 2.29]	0.997	3	1.04 [0.40, 2.74]	0.935
Normal	68	19	1.00 Ref	—	7	1.00 Ref	—
Normal testosterone (ng/mL) ACE‐27 comorbidity							
Moderate to severe	68	19	2.13 [1.19, 3.80]	0.01	7	2.07 [0.94, 4.56]	0.07
No or minimal	178	38	1.00 Ref	—	17	1.00 Ref	—
Low testosterone (ng/mL) ACE‐27 comorbidity							
Moderate to severe	27	9	1.12 [0.49, 2.58]	0.79	3	0.80 [0.30, 2.13]	0.65
No or minimal comorbidity	77	23	1.00 Ref	—	15	1.00 Ref	—
Interaction term: ACE‐27 comorbidity moderate to severe versus no or minimal × testosterone in ng/dL low versus normal	350	89	0.53 [0.19, 1.43]	0.21	42	0.39 [0.11, 1.30]	0.12
Age at randomization (years)	350	89	1.04 [1.01, 1.07]	0.02	42	1.00 [0.96, 1.04]	0.96
Gleason score							
9–10	125	48	2.14 [1.34, 3.43]	0.002	30	3.41 [1.73, 6.75]	< 0.001
8	42	7	0.66 [0.28, 1.54]	0.33	1	0.35 [0.04, 3.13]	0.35
7 or less	183	34	1.00 Ref	—	11	1.00 Ref	—
PSA in ng/mL							
> 20	94	31	1.53 [0.88, 2.66]	0.14	18	1.04 [0.50, 2.14]	0.92
> 10–20	95	21	0.92 [0.52, 1.63]	0.77	8	0.56 [0.23, 1.36]	0.20
< 4	27	8	1.42 [0.64, 3.15]	0.40	4	1.05 [0.34, 3.28]	0.94
≥ 4–10	134	29	1.00 Ref	—	12	1.00 Ref	—
Tumor category							
3b/4	33	14	1.68 [0.79, 3.56]	0.18	10	1.96 [0.64, 5.99]	0.24
2/3a	224	56	1.11 [0.63, 1.94]	0.72	26	0.93 [0.38, 2.31]	0.88
1c	93	19	1.00 Ref	—	6	1.00 Ref	—
Treatment							
RT and ADT and docetaxel	175	44	0.91 [0.59, 1.40]	0.66	22	1.04 [0.54, 2.00]	0.91
RT and ADT	175	45	1.00 Ref	—	20	1.00 Ref	—
Salvage ADT (*t*)	126	43	2.83 [1.72, 4.63]	< 0.001	36	19.41 [7.13, 52.84]	< 0.001

Abbreviations: ACE‐27: Adult Comorbidity Evaluation‐27 metric; ACM: all‐cause mortality; ADT: androgen deprivation therapy; AHR: adjusted hazard ratio; CI: confidence interval; ng/dL: nanograms/deciliter; ng/mL: nanograms/mL; No: number; PC: prostate cancer; PCSM: prostate cancer‐specific mortality; Ref: reference; RT: radiation therapy; *t*: time‐dependent; Yrs: years.

### 3.3 Adjusted Estimates of Prostate Cancer‐Specific and All‐Cause Mortality

**FIGURE 2:**
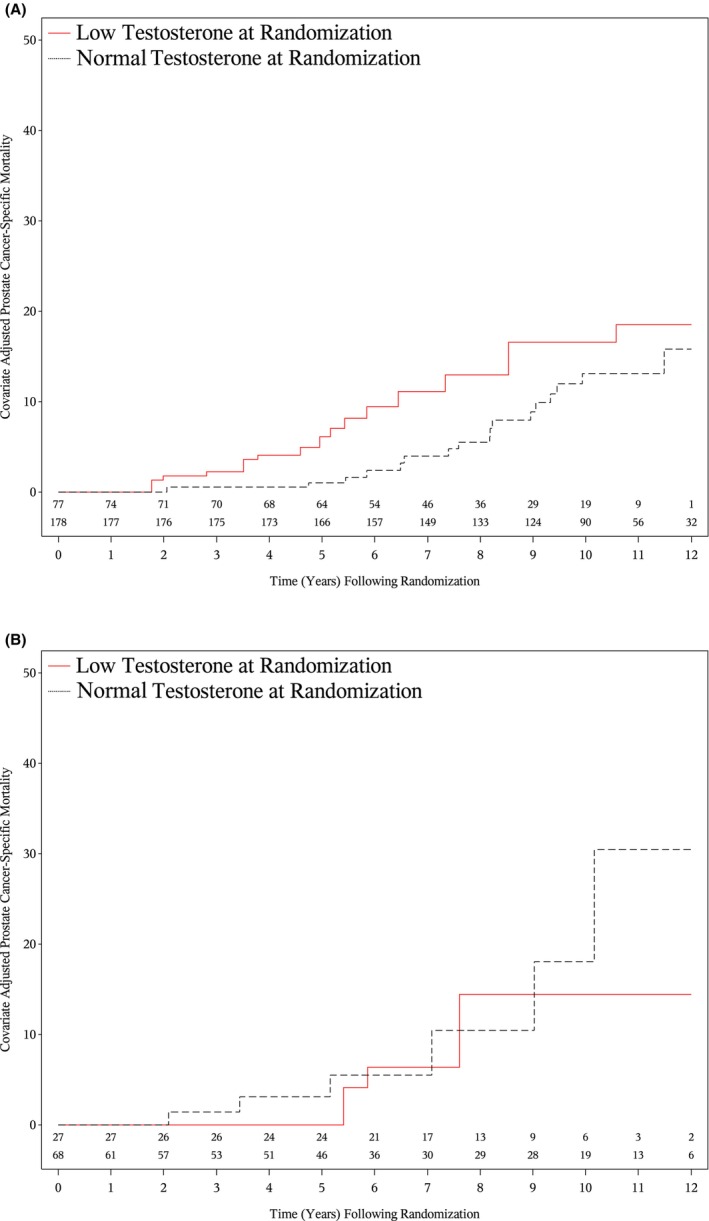
Adjusted estimates of prostate cancer‐specific mortality among patients with no or minimal (A), moderate to severe comorbidity (B) stratified by low versus normal testosterone level at randomization. Adjusted *p*‐value: no or minimal comorbidity: *p* = 0.01; moderate to severe comorbidity: *p* = 0.935.

**FIGURE 3:**
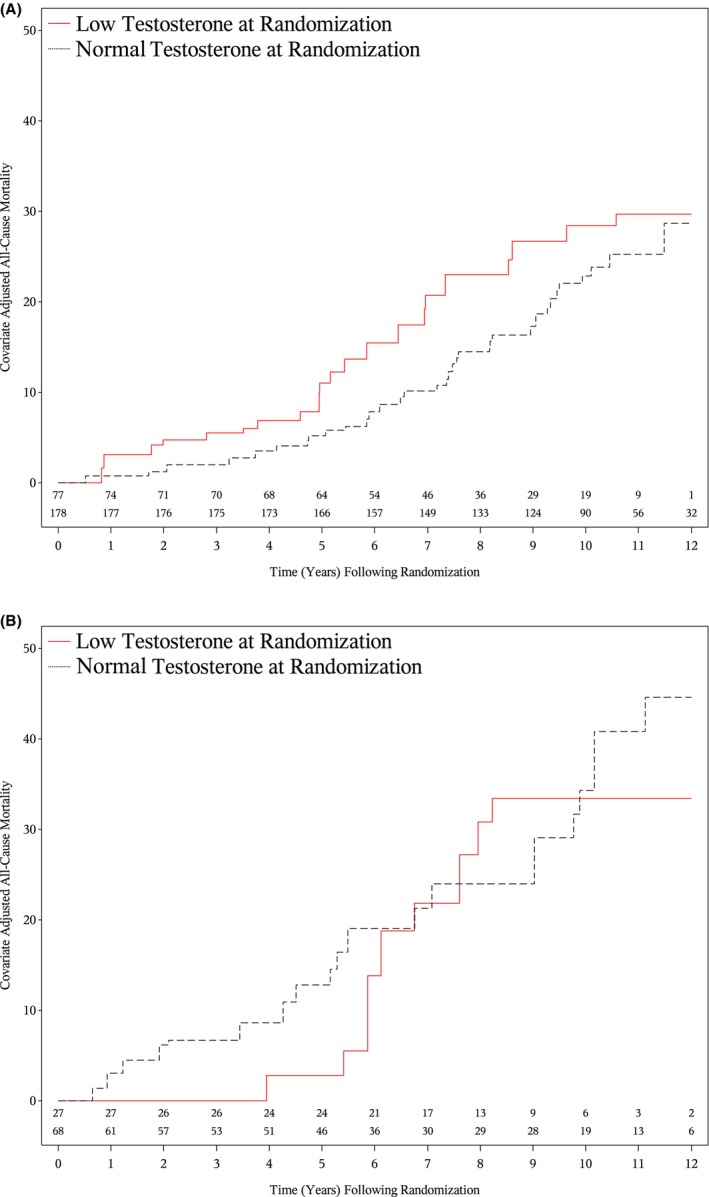
Adjusted estimates of all‐cause mortality among patients with no or minimal (A) moderate to severe comorbidity (B) stratified by low versus normal testosterone level at randomization. Adjusted *p*‐value: no or minimal comorbidity: *p* = 0.02; moderate to severe comorbidity: *p* = 0.997.

As shown in Figures 2A and 3A, higher adjusted estimates of PCSM (*p* = 0.01) and ACM (*p* = 0.02) were observed in patients with a low compared to normal testosterone level at randomization and no or minimal comorbidity, whereas this was not true among patients with moderate to severe comorbidity, as shown in Figures 2B and 3B (*p* = 0.935 and *p* = 0.997), respectively. Among patients with no or minimal comorbidity, 8‐year point estimates of PCSM were 12.93% (95% CI: 7.77%, 21.12%) for patients with a low testosterone level at randomization and 5.51% (95% CI: 2.85%, 10.52%) for those with a normal testosterone level at randomization. These respective estimates of ACM were 22.99% (95% CI: 15.88%, 32.60%) and 14.47% (95% CI: 9.88%, 20.92%). The analogous values for patients with moderate to severe comorbidity were 14.43% (95% CI: 5.86%, 33.14%) and 10.44% (95% CI: 4.18%, 24.79%) for PCSM and 30.83% (95% CI: 19.16%, 47.20%) and 23.97% (95% CI: 15.15%, 36.69%) for ACM.

## 4 Discussion

We found a significant association between low serum testosterone and advanced T‐category at diagnosis, and an increased PCSM and ACM risk in PSA‐screened patients who were in otherwise good health where patients with a normal testosterone at randomization served as the reference group. The clinical significance of this observation is that people with a low serum testosterone level undergoing PSA‐based screening for PC appear to be at increased risk of presenting with more advanced PC and a higher risk of dying from PC, resulting in an increased all‐cause mortality risk. Supporting this observation is a recent study showing GAHT use in transgender women was associated with a lower prevalence of PC, but also an increased risk of PSA recurrence and distant metastasis [36]. These observations can be explained by the blunting of PSA expression from the use of GAHT [23, 37] and as a result, when the PSA level finally exceeds 4 ng/mL, these patients have more advanced and less curable PC. Therefore, a prospective assessment of the impact of an alternative screening method on T‐category and Gleason score at presentation and PCSM is needed for people with low serum testosterone levels.

While a digital rectal examination (DRE) could be a consideration, it is subjective with inter‐examiner variability [20] and prior to the PSA era, patients presenting with an abnormal DRE had cancers that were more advanced than what we observe today with PSA‐based screening [38]. As a result, this option does not appear the best. Rather, mpMRI could be considered because, unlike the serum PSA level, the mpMRI images are not known to be influenced by the serum testosterone level. Specifically, cancer detection rates on targeted biopsy guided by mpMRI are not significantly different in patients with low versus normal testosterone levels [39]. Moreover, unlike the inter‐examiner variability observed with the DRE [20] using mpMRI, radiologists of different experience levels have excellent agreement for detecting index lesions, which are the targets for biopsy [40].

Several points deserve further consideration. First, it is possible that even with early detection using mpMRI, PCSM would not be decreased in men and transgender women with a low serum testosterone because even with earlier detection, a PC that develops in a low serum testosterone milieu may still be aggressive. Moreover, the biology of PC in these two populations may differ because transgender women on GAHT typically have testosterone levels < 50 ng/dL, whereas men in this study whose serum testosterone was low had a median value of 246 ng/dL. In addition, estrogen, which is part of GAHT, may impact the androgen responsiveness of PC that later develops [41] and it remains unknown whether testosterone replacement therapy (TRT), used in men with low testosterone given its detrimental impact on OS [11], can temper the aggressiveness of a subsequently diagnosed PC. However, these questions could be rigorously assessed in a prospective randomized screening trial. The trial design would include two screening arms: (1) a standard of care arm which involves an annual PSA assessment and pursuing mpMRI and then prostate biopsy only if the PSA is elevated, and (2) an investigational arm which involves performing an annual mpMRI and PSA assessment and pursuing prostate biopsy if either is abnormal. A pre‐randomization stratification could be employed, being transgender women on GAHT versus men with a low serum testosterone not on TRT and men whose low serum testosterone was corrected with TRT to assess the impact that early detection using mpMRI could have on the primary and secondary endpoints in these three populations. The study would be powered with the appropriate sample size to achieve this goal. The gold standard long‐term primary endpoint would be PCSM. Secondary endpoints would include T‐category and Gleason score at presentation with particular interest around the detection of clinically insignificant versus significant prostate cancer [42]. Second, whether there exists a PSA level or PSA increase over a year that should prompt ordering an mpMRI and biopsy to detect clinically significant PC earlier than a PSA exceeding the upper limit of normal in people with low serum testosterone remains unknown. However, answering this question would be challenging because of the variation in low testosterone levels between individuals and the androgen dependence of PSA expression [10]. This variation would make it difficult to identify an appropriate cut point for study. Third, prostate‐specific membrane antigen (PSMA) positron emission tomography (PET) imaging has been shown to be superior to mpMRI in detecting the dominant lesion in the prostate [43]. Therefore, questions of interest for future study would be whether PSMA PET imaging or genomic classifiers such as the 4K score or the prostate health index [44] designed to assess for the presence of any or aggressive PC, respectively, could enhance earlier detection of clinically significant PC in people with low serum testosterone levels. Yet, a significant clinical correlation between predefined genomic risk score groups and testosterone level groups at diagnosis has not been observed [45]. However, in the current study, we observe a significant association between increased PCSM and ACM risk in patients with no or minimal comorbidity with low as compared to normal testosterone at presentation. Taken together, these two observations suggest that it is the delay in diagnosis using PSA‐based screening in patients with a low testosterone level that leads to a worse long‐term outcome and not necessarily a more aggressive prostate cancer biology. Alternatively, it may be that the genes that portend a worse prognosis in patients who develop prostate cancer in the setting of a low serum testosterone have yet to be discovered and therefore were not included in the genomic risk metrics studied [45]. Future studies will be needed to elucidate the answer.

## 5 Conclusion

In conclusion, unlike PSA, multiparametric mpMRI images are not known to be influenced by the serum testosterone level; therefore, evaluating whether T‐category and Gleason score at presentation, and PCSM, can be reduced using mpMRI and PSA‐based versus PSA‐based screening alone in otherwise healthy men or transgender women with a low serum testosterone level should be considered.

## Author Contributions

**Sayeh Fattahi:** conceptualization (equal), investigation (equal), methodology (equal), writing – original draft (equal), writing – review and editing (equal). **Ming‐Hui Chen:** data curation (equal), formal analysis (equal), methodology (equal), software (equal), validation (equal), writing – original draft (equal), writing – review and editing (equal). **Jing Wu:** data curation (equal), formal analysis (equal), investigation (equal), methodology (equal), software (equal), writing – original draft (equal), writing – review and editing (equal). **Alicia C. Smart:** conceptualization (equal), formal analysis (equal), investigation (equal), methodology (equal), writing – original draft (equal), writing – review and editing (equal). **Anthony V. D'Amico:** conceptualization (equal), formal analysis (equal), investigation (equal), methodology (equal), project administration (lead), supervision (lead), writing – original draft (equal), writing – review and editing (equal).

## Conflicts of Interest

The authors declare no conflicts of interest.

## Supporting information


**Data S1:** cam471124‐sup‐0001‐DataS1.docx.

## Data Availability

Deidentified data used for the analysis in the current study can be made available upon request from the senior author (Dr. Anthony V D'Amico).
