# Supplementary material for: Low Testosterone Level and Mortality Risk in Patients With Prostate Cancer: A Post‐Randomization Analysis
Source: Cancer Med. 2025 Aug 1;14(15):e71124. doi: 10.1002/cam4.71124 (PMC12314635; doi:10.1002/cam4.71124)
Supplement: Supplementary file 1 — Data S1: cam471124‐sup‐0001‐DataS1.docx. [file CAM4-14-e71124-s001.docx]

SAS Codes for ACM Cox Regression

**proc** **phreg** data=comb2c;

title 'ACM Cox Regression';

model os*dead(**0**)=**cm23 TestoLownew TestoLo**wnewcm23 age GS8 GS9H psa4L

psa10_20 psa20H T23a T3b4 trt sADTt /rl;

TestoLownewcm23=TestoLownew*cm23;

sADTt=**0**;

if sADT=**1** and os>= sADTtime then sADTt=**1**;

cm23sADTt=cm23*sADTt;

cm23trt=cm23*trt;

**estimate 'TestoLownew for cm23=0' TestoLownew 1/exp cl;**

**estimate 'TestoLownew for cm23=1' TestoLownew 1 TestoLownewcm23 1/exp cl;**

**estimate 'cm23 for TestoLownew=0' cm23 1/exp cl;**

**estimate 'cm23 for TestoLownew=1' cm23 1 TestoLownewcm23 1/exp cl;**

**run**;

**SAS Output from Cox Regression**

| **Analysis of Maximum Likelihood Estimates** | | | | | | | | |
| --- | --- | --- | --- | --- | --- | --- | --- | --- |
| **Parameter** | **DF** | **Parameter Estimate** | **Standard Error** | **Chi-Square** | **Pr > ChiSq** | **Hazard Ratio** | **95% Hazard Ratio Confidence Limits** | |
| cm23 | 1 | 0.75483 | 0.29564 | 6.5188 | 0.0107 | 2.127 | 1.192 | 3.797 |
| TestoLownew | 1 | 0.64090 | 0.27596 | 5.3939 | 0.0202 | 1.898 | 1.105 | 3.260 |
| TestoLownewcm23 | 1 | -0.64227 | 0.50996 | 1.5862 | 0.2079 | 0.526 | 0.194 | 1.429 |
| **age** | 1 | 0.03677 | 0.01638 | 5.0408 | 0.0248 | 1.037 | 1.005 | 1.071 |
| **GS8** | 1 | -0.41999 | 0.43354 | 0.9385 | 0.3327 | 0.657 | 0.281 | 1.537 |
| **GS9H** | 1 | 0.76132 | 0.23997 | 10.0653 | 0.0015 | 2.141 | 1.338 | 3.427 |
| **psa4L** | 1 | 0.34721 | 0.40884 | 0.7212 | 0.3957 | 1.415 | 0.635 | 3.154 |
| **psa10_20** | 1 | -0.08681 | 0.29278 | 0.0879 | 0.7668 | 0.917 | 0.517 | 1.627 |
| **psa20H** | 1 | 0.42221 | 0.28306 | 2.2248 | 0.1358 | 1.525 | 0.876 | 2.656 |
| **T23a** | 1 | 0.10217 | 0.28648 | 0.1272 | 0.7214 | 1.108 | 0.632 | 1.942 |
| **T3b4** | 1 | 0.51585 | 0.38459 | 1.7991 | 0.1798 | 1.675 | 0.788 | 3.560 |
| **trt** | 1 | -0.09658 | 0.21994 | 0.1928 | 0.6606 | 0.908 | 0.590 | 1.397 |
| **sADTt** | 1 | 1.03881 | 0.25224 | 16.9611 | <.0001 | 2.826 | 1.724 | 4.633 |

**SAS output from 4 contracts under Cox regression**

| **Estimate** | | | | | | | | |
| --- | --- | --- | --- | --- | --- | --- | --- | --- |
| **Label** | **Estimate** | **Standard Error** | **z Value** | **Pr > \|z\|** | **Alpha** | **Lower** | **Upper** | **Exponentiated** |
| **TestoLownew for cm23=0** | 0.6409 | 0.2760 | 2.32 | 0.0202 | 0.05 | 0.1000 | 1.1818 | 1.8982 |

| **Estimate** | | |
| --- | --- | --- |
| **Label** | **Exponentiated Lower** | **Exponentiated Upper** |
| **TestoLownew for cm23=0** | 1.1052 | 3.2601 |

| **Estimate** | | | | | | | | |
| --- | --- | --- | --- | --- | --- | --- | --- | --- |
| **Label** | **Estimate** | **Standard Error** | **z Value** | **Pr > \|z\|** | **Alpha** | **Lower** | **Upper** | **Exponentiated** |
| **TestoLownew for cm23=1** | -0.00137 | 0.4242 | -0.00 | 0.9974 | 0.05 | -0.8328 | 0.8301 | 0.9986 |

| **Estimate** | | |
| --- | --- | --- |
| **Label** | **Exponentiated Lower** | **Exponentiated Upper** |
| **TestoLownew for cm23=1** | 0.4348 | 2.2935 |

| **Estimate** | | | | | | | | |
| --- | --- | --- | --- | --- | --- | --- | --- | --- |
| **Label** | **Estimate** | **Standard Error** | **z Value** | **Pr > \|z\|** | **Alpha** | **Lower** | **Upper** | **Exponentiated** |
| **cm23 for TestoLownew=0** | 0.7548 | 0.2956 | 2.55 | 0.0107 | 0.05 | 0.1754 | 1.3343 | 2.1273 |

| **Estimate** | | |
| --- | --- | --- |
| **Label** | **Exponentiated Lower** | **Exponentiated Upper** |
| **cm23 for TestoLownew=0** | 1.1917 | 3.7973 |

| **Estimate** | | | | | | | | |
| --- | --- | --- | --- | --- | --- | --- | --- | --- |
| **Label** | **Estimate** | **Standard Error** | **z Value** | **Pr > \|z\|** | **Alpha** | **Lower** | **Upper** | **Exponentiated** |
| **cm23 for TestoLownew=1** | 0.1126 | 0.4253 | 0.26 | 0.7912 | 0.05 | -0.7209 | 0.9461 | 1.1191 |

| **Estimate** | | |
| --- | --- | --- |
| **Label** | **Exponentiated Lower** | **Exponentiated Upper** |
| **cm23 for TestoLownew=1** | 0.4863 | 2.5756 |
